# Risk perception and communal coping in families affected by type 2 diabetes

**DOI:** 10.1093/geroni/igab046.2587

**Published:** 2021-12-17

**Authors:** Jielu Lin, Melissa Zajdel, Melanie Myers, Laura Koehly

**Affiliations:** 1 National Institutes of Health, Bethesda, Maryland, United States; 2 Cincinnati Children's Hospital Medical Center, Cincinnati, Ohio, United States

## Abstract

Despite a recent decline, rates of type 2 diabetes remain high among older adults. Preventing and delaying the onset of the condition with lifestyle changes is key to reducing disease burden in the population. Type 2 diabetes is a complex disease, likely a result from the joint effect of genetic, socio-environmental and lifestyle risk factors that are clustered in families. As such, the prevention of type 2 diabetes is a communal coping process, where individuals communicate about risk and establish routines to facilitate one another’s health habits and compliance with therapeutics. This poster investigates how such a process is affected by one’s perception of risk based on his/her knowledge about family health history (FHH). We collected family network data from families of different racial backgrounds in the greater Cincinnati area (28 white and 17 black/ African American households; 127 participants). The analysis focuses on how the density of diabetes diagnosis in one’s FHH affects communication about shared risk for type 2 diabetes and encouragement to maintain or adopt a healthy lifestyle. Results suggest a higher concentration of diabetes diagnosis in one’s FHH is associated with a higher number of risk communication ties in all families. With regards to encouragement ties, high rates of diabetes diagnosis in FHH are associated with an increased number of encouragement ties only in families of black/African heritage. The findings highlight the need and promise of using FHH to motivate co-encouragement to maintain/adopt a healthier lifestyle in families of black/African heritage.

